# Profile of Phosphatidylserine Modifications under Nitroxidative Stress Conditions Using a Liquid Chromatography-Mass Spectrometry Based Approach

**DOI:** 10.3390/molecules24010107

**Published:** 2018-12-29

**Authors:** Bruna Neves, Pedro Domingues, Maria Manuel Oliveira, Maria do Rosário Domingues, Tânia Melo

**Affiliations:** 1 Mass Spectrometry Centre, UI QOPNA, Chemistry Department, University of Aveiro, 3810-193 Aveiro, Portugal; brunafbneves@gmail.com (B.N.); p.domingues@ua.pt (P.D.); mrd@ua.pt (M.d.R.D.); 2 Chemistry Department, University of Trás-os-Montes e Alto Douro, 5000-801 Vila Real, Portugal; mmso@utad.pt; 3 Biology Department & CESAM & ECOMARE, University of Aveiro, 3810-193 Aveiro, Portugal

**Keywords:** lipidomic, nitration, nitroxidative stress, phosphatidylserine, tandem mass spectrometry, LC-MS

## Abstract

Nitrated lipids have been detected in vitro and in vivo, usually associated with a protective effect. While nitrated fatty acids have been widely studied, few studies reported the nitration and nitroxidation of the phospholipid classes phosphatidylcholine, and phosphatidylethanolamine. However, no information regarding nitrated and nitroxidized phosphatidylserine can be found in the literature. This work aims to identify and characterize the nitrated and nitroxidized derivatives of 1-palmitoyl-2-oleoyl-*sn*-3-glycero-phosphoserine (POPS), obtained after incubation with nitronium tetrafluoroborate, by liquid chromatography (LC) coupled to mass spectrometry (MS) and tandem MS (MS/MS). Several nitrated and nitroxidized products were identified, namely, nitro, nitroso, nitronitroso, and dinitro derivatives, as well as some nitroxidized species such as nitrosohydroxy, nitrohydroxy, and nitrohydroperoxy. The fragmentation pathways identified were structure-dependent and included the loss of HNO and HNO_2_ for nitroso and nitro derivatives, respectively. Combined losses of PS polar head group plus HNO or HNO_2_ and carboxylate anions of modified fatty acyl chain were also observed. The nitrated POPS also showed antiradical potential, demonstrated by the ability to scavenge the ABTS^●+^ and DPPH^●^ radicals. Overall, this in vitro model of nitration based on LC-MS/MS provided additional insights into the nitrated and nitroxidized derivatives of PS and their fragmentation fingerprinting. This information is a valuable tool for targeted analysis of these modified PS in complex biological samples, to further explore the new clues on the antioxidant potential of nitrated POPS.

## 1. Introduction

Phosphatidylserine (PS) is a key phospholipid of the inner leaflet of the cell membranes, and it also participates in several signaling and biological processes, namely targeting and function of intracellular signaling proteins [1,2,3]. However, PS and also oxidized PS can be translocated to the outer leaflet and be exposed at the cell surface, in early stages of apoptosis, which is a well-known signaling of apoptotic cells [3,4,5]. Identification of oxidized PS in biological samples, as well as its biological role, has been the outcome of several studies that used mass spectrometry-based approaches, allowing to detected oxygenated PSs both in vitro and in vivo [6,7,8,9]. Depending on the oxidation product and the biological context, PS oxidation products are reported to have both anti and/or pro-inflammatory actions [10,11]. However, in physiological and pathological conditions, reactive nitrogen species are also responsible for the nitration and nitroxidation of biomolecules, including lipids. Several studies identified and quantified nitrated fatty acids in biological systems, based on advanced mass spectrometry strategies, allowing to pinpoint nitrated fatty acids as important signaling molecules in health and disease [12,13,14]. Phospholipid nitration has also been reported in few in vitro mimetic models of nitroxidative stress conditions using phospholipid standards of phosphatidylcholines (PCs) and phosphatidylethanolamines (PEs) [15,16]. Nitrated and nitroxidized derivatives of PCs and PEs were also identified in vivo, namely in cardiac mitochondria isolated from the heart of an animal model of type 1 diabetes mellitus [15], and in vitro, in cardiomyoblast H9c2 cells under starvation conditions [16]. Moreover, recently, it was reported the potential of nitrated 1-palmitoyl-2-oleoyl-*sn*-glycero-3-phosphocholine as antioxidant and anti-inflammatory agent [17]. In spite of the work done for PC and PE nitrated derivatives, there is a lack of knowledge regarding the identification of nitrated and nitroxidized derivatives of PS by mass spectrometry. This needs to be overcome since the identification of specific fragmentation pathways, and reporter ion of these modified PS, is essential to detect these modified lipids in biological samples and to screen their putative biological role. Driven by the lack of knowledge on phospholipid nitration, particularly on PS nitrated and nitroxidized products, in this study, 1-palmitoyl-2-oleoyl-*sn*-glycero-3-phosphoserine (POPS, PS16:0/18:1) was incubated with nitronium tetrafluoroborate (NO_2_BF_4_), and the new nitrated and nitroxidized products were studied by LC-MS approaches. Nitronium tetrafluoroborate is a salt of nitronium ion (NO_2_^+^) that is a well-known mimetic nitrating agent used in previous studies on phospholipid [15], and fatty acid nitration [12]. POPS was selected because it is one of the abundant PS molecular species detected in some cell types as keratinocytes [18], and tissues as intestinal and lung [19]. Additionally, oleic acid seems to be one of the targets of nitration [20] and nitro-oleic acid is considered a bioactive lipid with anti-inflammatory [21,22], and antioxidant properties [23]. Identification of nitrated/nitroxidized PS species was performed by electrospray ionization mass spectrometry (ESI-MS) and reversed-phase liquid chromatography (LC-MS). Nitrated and nitroxidized PS species were identified and structurally characterized by tandem mass spectrometry using higher collisional (HCD) and low collision-induced dissociation (CID) methods. The radical scavenging capacity of nitrated POPS against 2,2-diphenyl-1-picrylhydrazyl (DPPH^●^) and 2,2-azino-*bis*-3-ethylbenzthiazoline-6-sulphonic acid (ABTS^●+^) radicals were also evaluated. 

## 2. Results and Discussion

**Figure 1:**
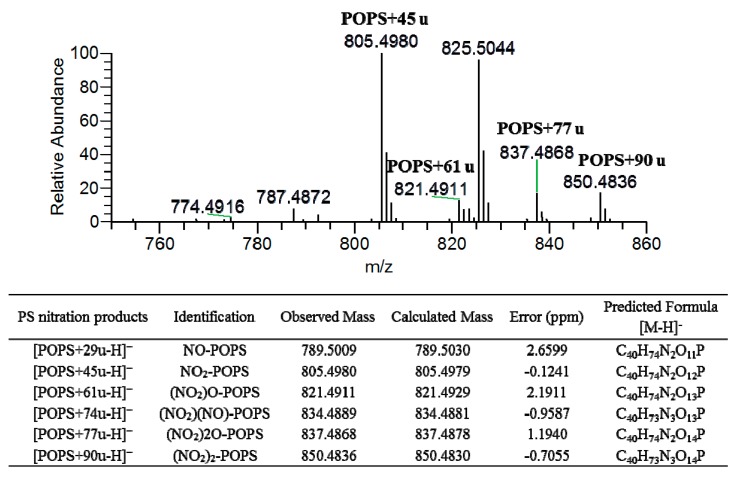
ESI-MS spectrum of POPS after nitration reaction acquired in the negative-ion mode in Q-Exactive Orbitrap. Assignments of nitrated and nitroxidized derivatives formed after reaction between NO_2_BF_4_ and POPS observed in the ESI-MS spectrum as [M − H]^−^ ions were confirmed by mass accuracy. The calculated and observed mass, error, and formula of the nitrated and nitroxidized derivatives formed after reaction between NO_2_BF_4_ and POPS observed in the ESI-MS spectrum are also shown. Error (ppm) = (Observed *m/z* − Calculated *m/z*)/Calculated *m/z*) × 1 × 10^6^.

**Figure 2:**
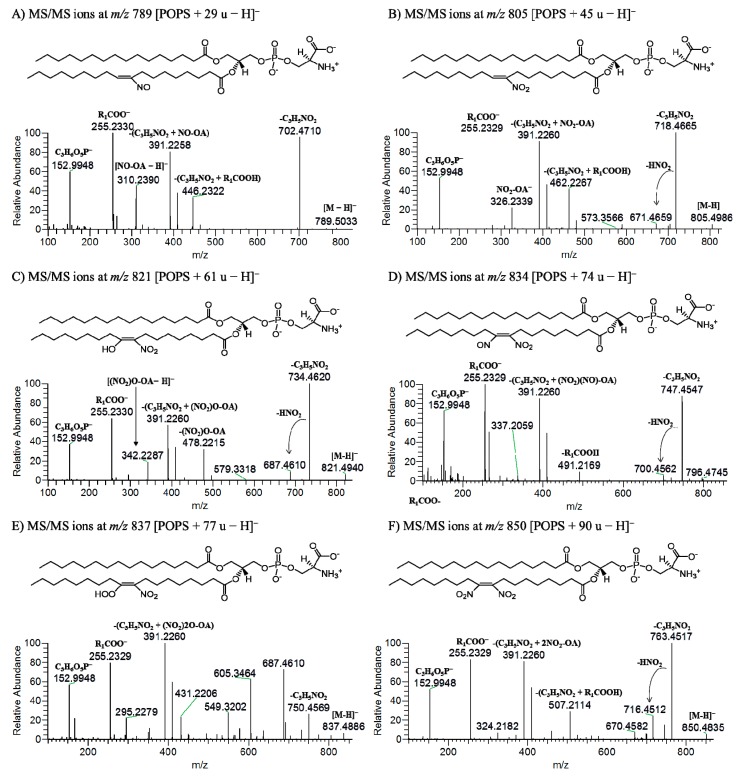
ESI-MS/MS spectra obtained in Q-Exactive Orbitrap of [M − H]^−^ ions of nitroso, nitrated and nitroxidized POPS at *m/z* 789.5009 ([POPS + 29 u − H]^−^) assigned as NO-POPS (**A**), at *m/z* 805.4980 ([POPS + 45 u − H]^−^) assigned as NO_2_-POPS (**B**), at *m/z* 821.4911 ([POPS + 61 u − H]^−^) assigned as (NO_2_)O-POPS (**C**), at *m/z* 834.4889 ([POPS + 74 u − H]^−^) assigned as (NO_2_)(NO)-POPS (**D**), at *m/z* 837.4868 ([POPS + 77 u − H]^−^) assigned as (NO_2_)2O-POPS (**E**), and at *m/z* 850.4836 ([POPS + 90 u − H]^−^) assigned as (NO_2_)_2_-POPS (**F**). One possible chemical structure of each deprotonated molecule is also proposed, with the nitro group located in C9, but other possibilities should be considered, namely the nitro group at C10, as reported for the nitration of oleic acid [12].

In this study, the nitration of POPS was induced in a mimetic system of nitration by incubation with nitronium tetrafluoroborate (NO_2_BF_4_). The reaction occurred in hydrophobic environments, mimicking the nitration that occurs in biological membranes [24], where reactive nitrogen species (RNS) easily diffuse and accumulate in the phospholipid bilayers [25,26,27]. NO_2_BF_4_, a salt of nitronium ion (NO_2_^+^), has been used to induce the nitration of phospholipids [15,16] and fatty acids [12]. It was previously reported that NO_2_BF_4_ is able to mimic the nitration induced by peroxynitrite and nitrite [28], and the nitrated lipids formed under these conditions were described to be similar to those identified in biological samples showing the same fragmentation pattern as nitrated lipids found in plasma and lipoproteins [28]. The formation of nitrated and nitroxidized products of POPS after reaction with NO_2_BF_4_ was monitored by ESI-MS in the negative-ion mode in a Q-Exactive Orbitrap (Figure 1). The nitrated and nitroxidized PS products were identified as [M − H]^−^ ions, as summarized in Table 1. These products were assigned as nitroso (NO-PS, POPS + 29 u) at *m/z* 789.5009, the most abundant product nitro (NO_2_-PS, POPS + 45 u) at *m/z* 805.4980, dinitro ((NO_2_)_2_-PS, POPS + 90 u) at *m/z* 850.4836, and nitronitroso ((NO_2_)(NO)-PS, POPS + 74 u) derivatives at *m/z* 834.4889. Nitroxidized PSs, namely nitrohydroxy ((NO_2_)O-PS, POPS + 61 u) at *m/z* 821.4911, and nitrohydroperoxy ((NO_2_)2O-PS, POPS + 77 u) at *m/z* 837.4868 were also identified. These type of modifications have been reported in the previous studies of modifications of PCs and PEs by RNS [16]. The nitrated and nitroxidized PS derivatives were assigned based on the mass shift compared with the unmodified POPS, as well as the accurate mass measurement and elemental composition determination (Figure 1). Tandem mass spectra (Figure 2 and Table 1) were analyzed in order to pinpoint the specific fragmentation fingerprinting and the typical reporter ions of each modification. All the product ions displayed in Table 1 are coming from higher CID (HCD) fragmentation obtained in the high resolution Q-Exactive Orbitrap, which were also confirmed and assigned based on accurate mass measurement. MS/MS spectra were obtained using low CID and higher CID (HCD), respectively, using low-resolution Linear Ion Trap (LIT) and in a high-resolution Orbitrap mass spectrometers. The analysis in a LIT also allowed performing MS^n^ experiments. Reversed-phase LC-MS and MS/MS experiments were also implemented in order to confirm the proposed assignments and to unveil the presence of possible isomeric structures.

**Figure 3:**
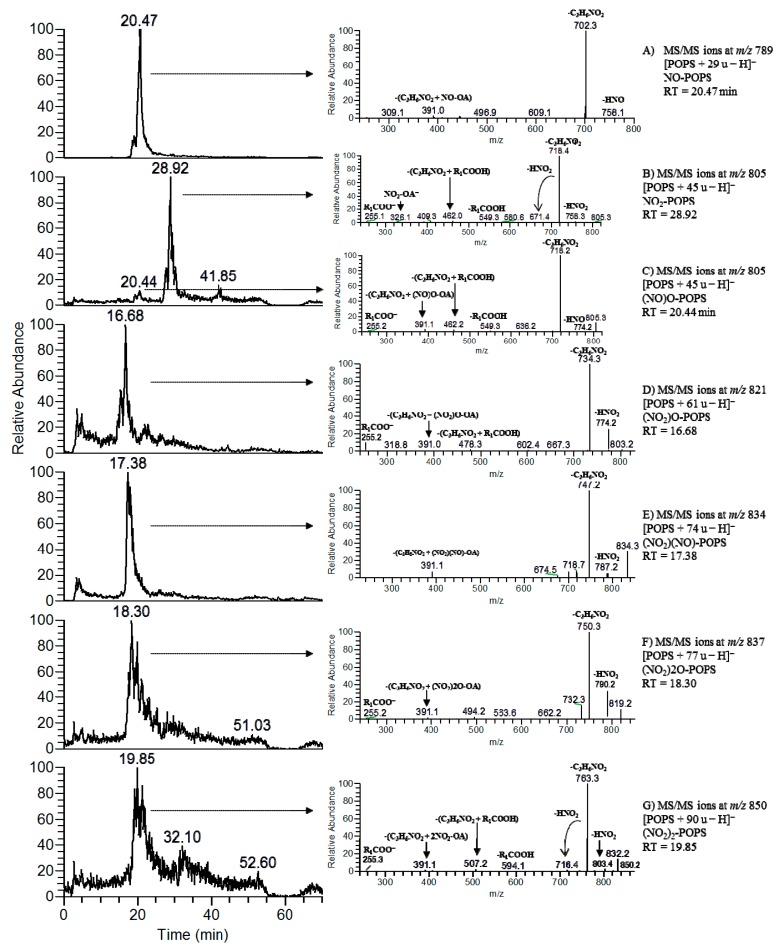
Reconstructed ion chromatogram (RIC) and LC-MS/MS spectra (**A**–**G**) of nitrated and nitroxidized derivatives of POPS at *m/z* 789 identified as nitroso POPS (RT 20.47 min) (**A**), isobaric compounds at *m/z* 805 identified as nitro (RT 28.92) (**B**) and nitrosohydroxy POPS (20.44 min) (**C**), at *m/z* 821 identified as nitrohydroxy POPS (RT 16.68 min) (**D**), at *m/z* 834 identified as nitronitroso POPS (RT 17.38 min) (**E**), at *m/z* 837 identified as nitrohydroperoxy POPS (RT 18.30 min) (**F**), and at *m/z* 850 identified as dinitro POPS (RT 19.85 min) (**G**).

The nitroso derivative (NO-POPS) displayed a mass shift of plus 29 u in comparison with the native POPS. In the tandem mass spectrum (MS/MS) of the [M − H]^−^ ion of NO-POPS at *m/z* 789.3, acquired in the LIT instrument, it was possible to observe the product ion at *m/z* 758.3, formed due to the loss of HNO, but no neutral loss of nitrous acid (HNO_2_) was observed (Figure S1A). Product ion identified as R_2_COO^−^, corresponding to the modified fatty acyl chain (NO-OA), were observed at *m/z* 310.2. The observed neutral loss of 87 u (ions at *m/z* 702.3) confirmed the presence of the serine polar head. The MS^3^ spectrum of the precursor ions at *m/z* 702.3 also showed the typical neutral loss of HNO (*m/z* 671.3) and the carboxylate anions of fatty acyl chain (*m/z* 310.2). It was not possible to observe the loss of HNO from the modified fatty acyl chain in both MS/MS and MS^3^ spectra. Tandem mass spectrum of the precursor ions at *m/z* 789.5033, acquired in the Orbitrap instrument, revealed the product ions formed by neutral loss of 87 at *m/z* 702.4701, the carboxylate anion of NO-OA at *m/z* 310.2382, and also the neutral loss of HNO from NO-OA at *m/z* 279.2324 (Figure 2A). After LC-MS analysis, the reconstructed ion chromatogram (RIC) of [NO-POPS − H]^−^ showed only one peak, suggesting the presence of only one modified species (Figure 3A). The MS/MS spectrum obtained at the retention time (RT) of 20.47 min showed the neutral loss of HNO, confirming the presence of the nitroso derivative.

Nitro (or nitroalkene) derivatives of PS displayed a mass shift of 45 u. The MS/MS spectra of the precursor ion at *m/z* 805.2 in LIT (Figure S1B) and *m/z* 805.4986 in Orbitrap (Figure 2B), showed ions formed due to the neutral loss of 87 u, at *m/z* 718.4665, arising from the loss of serine head group, the typical neutral loss of 47 u (-HNO_2_) at *m/z* 758.4982, the neutral loss of 134 u (combined neutral loss of 87 u + 47 u) at *m/z* 671.4659, and R_2_COO^−^ product ions of modified oleic acid (NO_2_-OA at *m/z* 326.2339 and (NO_2_-OA)-HNO_2_ at *m/z* 279.2330). Comparing the MS/MS spectra obtained from low CID and HCD, the same product ions were observed, but the neutral loss of 134 u (combined loss of 87 u + 47 u) and the carboxylate anions [NO_2_-OA − H]^−^ and [(NO_2_-OA) − HNO_2_ − H]^−^ were more abundant in HCD MS/MS spectrum. These ions can be selectively used as reported ions and for neutral loss scanning in order to identify the NO_2_-PS in biological samples. Nevertheless, the MS^3^ data at of the product ion at *m/z* 805.2→*m/z* 718.3 (NO_2_-POPS − 87 u), acquired in the LIT (Figure S1B1), also showed product ion formed by the neutral loss of HNO_2_ at *m/z* 671.3, and the carboxylate anions of NO_2_-OA at *m/z* 326.1 and (NO_2_-OA) − HNO_2_ at *m/z* 279.2, confirming the presence of the nitro group. In the LC-MS experiment, the ions at *m/z* 805.2 eluted in two different peaks, a major one that eluted latter at RT of 28.9 min and that corresponded to NO_2_-POPS, and a minor one that eluted earlier at RT of 20.4 min, which was assigned as (NO)O-POPS, an isobaric and structural isomeric species of NO_2_-POPS. The LC-MS/MS spectrum of the ion at *m/z* 805.3, acquired at RT of 20.44 min (Figure 3C), showed ion formed due to the neutral loss of HNO at *m/z* 774.2, but no neutral loss of HNO_2_ was observed, indicating the presence of a nitroso derivative and confirming the proposed assignment. On the other hand, the LC-MS/MS spectrum obtained at RT of 28.92 min (Figure 3B) showed a product ion at *m/z* 758.3, corresponding to the neutral loss of HNO_2_, and thus confirming the presence of a nitro group and the presence of a nitro derivative (NO_2_-POPS). Therefore, modified species eluting at a RT of 20.44 min were assigned as nitrosohydroxy derivatives ((NO)O-POPS), as previously reported for nitrated and nitroxidized PC and PE [16]. Nevertheless, the nitro derivative of POPS is clearly present, with a higher relative abundance.

Other nitrated PS product with a mass shift of plus 74 u, identified at *m/z* 834.6 in LIT and *m/z* 834.4883 in Orbitrap instruments, was assigned as nitronitroso derivatives ((NO_2_)(NO)-POPS). The MS/MS spectra obtained in both instruments showed product ions corresponding to loss of serine polar head (neutral loss of 87 u), neutral loss of 47 u (-HNO_2_), neutral loss of 134 u (87 u + 47 u), the carboxylate anions of nitronitroso oleic acid ((NO_2_)(NO)-OA), and the neutral loss of HNO_2_ from (NO_2_)(NO)-OA (Figure 3D, Figure S2 A,A1). These ions eluted in one peak (RT of 17.38 min) and the LC-MS/MS spectrum showed the neutral loss of HNO_2_ (Figure 3E). 

We also found a product formed due to the insertion of two nitro groups with a mass shift of plus 90 u (*m/z* 850.4835 in Orbitrap and *m/z* 850.3 in LIT). The MS/MS spectrum acquired in Orbitrap (Figure 2F) showed ions formed due to the loss of serine polar head (neutral loss of 87 u) at *m/z* 763.4517, neutral loss of HNO_2_ at *m/z* 803.4825, and neutral loss of serine polar head combined with the loss of one (134 u (neutral loss of 87 u + 47 u)) and two HNO_2_ molecules (181 u (neutral loss of 87 u + 94 u)) at *m/z* 716.4513 and 669.4505, respectively. These two consecutive neutral losses of HNO_2_ confirmed the presence of two nitro groups and hence the presence of a PS dinitro derivative. Carboxylate anions of dinitro oleic acid ((NO_2_)_2_-OA) were also detected at *m/z* 371.2191, together with product ions formed due to the neutral loss of one and two HNO_2_ from the (NO_2_)_2_-OA at *m/z* 324.2182 and 277.2174, respectively. MS/MS and MS^3^ spectra obtained in LIT also showed product ions formed by the two consecutive neutral losses of HNO_2_ and thus confirming the presence of the dinitro derivative (Figure S2B–B2). Major differences between MS/MS spectra from LIT and Orbitrap are related with the abundance of carboxylate anions of modified fatty acyl chain and product ions arising from combined losses of HNO_2_ and serine polar head, which are more abundant in the case of HCD, in opposition to ions arising from the loss of HNO_2_ that are present with higher abundance in CID. This modified PS eluted in one peak (RT of 19.85 min), and the LC-MS/MS spectrum showed the neutral loss of HNO_2_, confirming the presence of only one species (Figure 3G).

Nitrohydroxy (Figure 2C, Figure S3A–A3) and nitrohydroperoxy derivatives (Figure 2E, Figure S3B–B2) of POPS were also observed and characterized by tandem mass spectrometry. The typical fragmentation pathway of these species involves the neutral loss of serine polar head, the typical neutral loss of 47 u (HNO_2_), and the neutral loss of HNO_2_ combined with loss of serine head group. Carboxylate anions of modified oleic acid and product ions formed by neutral loss of HNO_2_ and O_2_ (only for nitrohydroperoxy derivative) from the modified oleic acid were also observed. Each derivative eluted in one peak (RT of 16.68 and 18.30 min, respectively) and their LC-MS/MS spectra also showed the neutral loss of HNO_2_.

**Figure 4:**
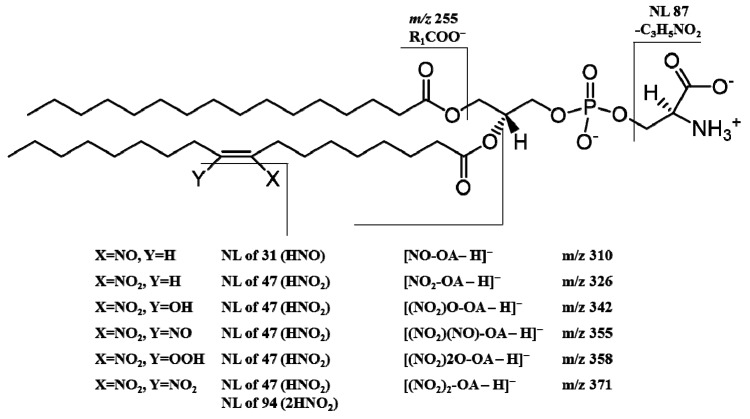
Schematic representation of major fragmentation pathways of nitrated POPS derivatives observed in negative-ion mode. A possible chemical structure of nitrated POPS with the nitro (NO_2_) and nitroso (NO) groups located in C9 is represented, but other possibilities should be considered, namely the nitro (NO_2_) and nitroso (NO) groups at C10, as reported for the nitration of oleic acid [12].

Comparing the information displayed in MS/MS data from CID and HCD, the major observed differences were related with different relative abundance of specific product ions. In general, the typical reporter ions for the identification of nitrated and nitroxidized POPS, that corresponds to the formation of product ions with higher *m/z* values, seems to be present in high relative abundance in MS/MS spectra from LIT, while low *m/z* reporter ions, namely the carboxylate anions correspondent to the modified fatty acyl chain, can be seen with a higher relative abundance in MS/MS spectra acquired in the Orbitrap. These variations are related to the different methods of ion activation during MS/MS experiments. It is well-.known that HCD (used in Orbitrap instruments) is able to enhance the yield of low molecular weight fragment ions due to the multiple collisions of both precursor and fragments ions with the gas molecules [29]. Thereby, it becomes essential to disclose the most relevant fragmentation pathways obtained when using different MS platforms and define the most useful neutral losses and reporter ions for accurate identification of these modified lipid species in complex biological matrices [30]. Since high-resolution instruments with Orbitrap technology that use as fragmentation method HCD are now being popular in lipidomic-based approaches, the detailed characterization of the fragmentation patterns of nitrated and nitroxidized PS becomes essential. The typical fragmentation profile provided with the mimetic model used in this study (summarized in Table 2 and Figure 4) can be used for the identification of these modified phospholipids in complex biological matrices, as already reported for mimetic models of nitration of PCs and PEs [15,16]. 

Previous results showed that nitrate POPC could have health protective effects as an antioxidant due to its capability of trapping harmful radicals [17], since it was able to scavenge the ABTS^●+^, DPPH^●^, and oxygen-derived radicals (ORAC assay), and protect against lipid peroxidation induced by the hydroxyl radical [17]. In this study, we have further evaluated the ability of non-modified POPS and nitrated POPS to trap radicals as H-atom donator and thus act as a scavenger of radicals, by performing experiments with DPPH^●^ and ABTS^●+^ radicals. Both assays were performed using all the nitration reaction mixture of POPS.

**Figure 5:**
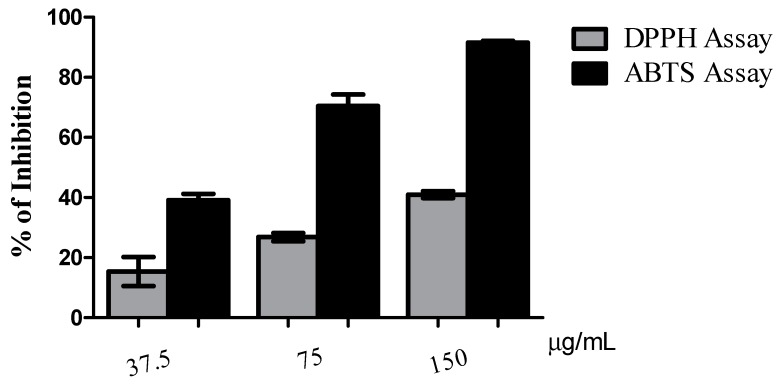
Percentage of inhibition of DPPH^●^ and ABTS^●+^ radicals remaining after 120 min of reaction in the presence of nitrated POPS at the three concentrations tested (37.5, 75 and 150 µg/mL in ethanol).

To evaluate the antioxidant activity of nitrated POPS, the decrease of absorbance at 517 nm and 734 nm for DPPH^●^ and ABTS^●+^ radicals, respectively, was measured and was expressed as the variation of the amount of both radicals (in percentage). The percentage of inhibition was calculated for each assay, and both DPPH^●^ and ABTS^●+^ radicals were reduced in the presence of nitrated POPS (Figure 5). For non-modified POPS, only a slight decrease of absorbance was observed at 517 nm and 734 nm for DPPH^●^ and ABTS^●+^ radicals, respectively, suggesting that non-modified POPS has lower potential as radical scavenger (Figure S4). This decrease was concentration-dependent, and the higher the amount of nitrated POPS, the higher the inhibition of DPPH^●^ and ABTS^●+^ radicals, and therefore the lower the percentage of radicals remaining after the reaction time. For ABTS^●+^ assay, the IC_50_ value was 48.71 µg/mL and the TE of 429.35 µmol Trolox/ g of nitrated POPS. For DPPH^●^ assay, we were not able to calculate the IC_50_ value since the percentage of inhibition was lower than 50%, and thus we calculated the IC_25_ value, which was 75.16 µg/mL while the TE value was 185.82 µmol Trolox/ g of nitrated POPS. These results suggest that nitrated POPS has higher reactivity and thus higher H-atom donating ability in the aqueous medium than in organic environments. This is in agreement with previous information that also states that NO_2_-FA decay faster in aqueous medium [31] than in hydrophobic environments as the biological membranes, where they are hydrophobically stabilized. This high reactivity of nitrated POPS in hydrophilic media can be related with its biological roles in physiologically relevant conditions, which can also occur via formation of other biologically relevant species [32]. Additionally, these results are similar with the ones reported for nitrated POPC in the same conditions, which is quite interesting because nitrated POPC was also reported with anti-inflammatory activity [17]. Nevertheless, the ability of nitrated POPS to quench the DPPH^●^ and ABTS^●+^ radicals was higher when comparing with the data from nitrated POPC, which was demonstrated by the lower IC values and higher TE for nitrated POPS [17]. In the literature, it was reported that the primary amine moiety of PS could act as an electron donator rather than the tertiary amine moiety of PC, which allowed PS to be a more effective scavenger of lipid peroxyl radicals [33]. Also, it was reported that NO_2_ moiety of the NO_2_-OA accumulates near the phosphate group of phospholipid polar head, at the membrane-water interface. This accumulation shields the NO_2_ moiety from the hydrophobic environments, where NO_2_-FA exist in a stabilized form, making it more reactive [34]. Moreover, H-atom donating ability was previously described for NO_2_-FA [32]. The synergic relationship between PS polar head group, acting as an active electron donator, and NO_2_ moiety, acting as an H-atom donator, can be related with the apparent high antioxidant potential of nitrated POPS, when compared with nitrated POPC. The ability of nitrated PS to act as scavenger could be interesting at the biological level also due to the combination of different mechanisms of antioxidant (inter)actions. The anionic polar head group of PS can also display antioxidant actions through the ability of chelating iron ions and preventing the decomposition of hydroperoxides, reducing the lipid peroxidation chain reaction [35,36]. 

Altogether, the results gathered with this study highlight the need to keep up with the research in the field of phospholipid nitration, improving the detection of these species and contributing to the understanding of their biological roles. 

## 3. Materials and Methods

### 3.1. Reagents/Chemicals

The 1-palmitoyl-2-oleoyl-*sn*-glycero-3-phospho-l-serine (POPS) standard was purchased from Avanti^®^ Polar Lipids, Inc. (Alabaster, AL, USA) and used without further purification. Nitronium tetrafluoroborate (NO_2_BF_4_) was purchased from Sigma-Aldrich (Madrid, Spain). Perchloric acid 70% was purchased from Panreac (Barcelona, Spain). Ammonium molybdate and sodium dihydrogen phosphate dehydrated (NaH_2_PO_4_·2H_2_O) were purchased from Riedel-de Haёn (Seelze, Germany). Ascorbic acid was purchased from VWR International (Leicestershire, UK). HPLC grade chloroform, methanol and acetonitrile were purchased from Fisher Scientific Ltd. (Leicestershire, UK). Milli-Q water was filtered through a 0.22-mm filter and obtained using a Milli-Q Millipore system (Synergy^®^, Millipore Corporation, Billerica, MA, USA). 

### 3.2. Nitration of Phosphatidylserines

Phosphatidylserine nitration was carried out with nitronium tetrafluoroborate (NO_2_BF_4_), as previously described [15,16]. A solution of POPS (1 mg) in chloroform (1 mL) was prepared in an amber vial tube, and an excess of the solid NO_2_BF_4_ (≈1 mg) was added. The reaction mixture was incubated at room temperature for 1h, under orbital shaking at 750 rpm. After incubation, the mixture was transferred to a centrifuge glass tube, and the reaction was stopped by solvent extraction with Milli-Q water to hydrolyze unreacted NO_2_BF_4_ and/or to separate contaminating anions (such as nitrite (NO_2_^−^), nitrate (NO_3_^−^), and the tetrafluoroborate anion (BF_4_^−^)) from phospholipids [15,16,24]. The mixture was vortexed for 30 s and then centrifuged at room temperature at 2000 rpm for 10 min using a Mixtasel Centrifuge (Selecta, Barcelona, Spain). The organic layer containing the nitrated and nitroxidized derivatives of POPS was collected, evaporated under a nitrogen stream, and stored at −20 °C to be further quantified using a phosphorous assay [37], and analyzed by ESI-MS and reverse phase liquid chromatography coupled to ESI-MS (C5-RP-LC-MS) and MS/MS.

### 3.3. Phosphorous Measurement-Phospholipid Quantification

The total amount of nitrated and nitroxidized products of POPS recovered after extraction was quantified with the phosphorus assay, as previously described by Bartlett and Lewis [37], with modifications. Briefly, 125 µL of perchloric acid (70% *m/v*) was added to 10 µL of nitrated POPS (stock-solution was dissolved in 1 mL of CHCl_3_). The solution was dried under a nitrogen stream before the addition of perchloric acid. The nitrated POPS was then incubated for 1 h at 180 °C in the heating block (Stuart^®^, London, UK). Afterward, 825 µL of H_2_O, 125 µL of 2.5% ammonium molybdate (*m/v*; 2.5 g of NaMoO_4_·H_2_O in 100 mL of Milli-Q H_2_O) and 10% ascorbic acid (*m/v*; 0.1 g in 1 mL Milli-Q H_2_O) were added with homogenization in a vortex mixer after each addition. Then, the nitrated POPS was incubated for 10 min at 100 °C in a water bath. Standards from 0.1 to 2 µg of phosphate (NaH_2_PO_4_·2H_2_O, 100 μg of phosphorus per mL) underwent the same sample treatment, with the exception of the heating block phase. A volume of 200 µL of each standard and nitrated POPS were added to the 96-well multiwell plate, and the absorbance was measured at 797 nm in a Multiskan GO Microplate Spectrophotometer (Thermo Scientific, Hudson, NH, USA)). The amount of phosphorus was calculated by linear regression analysis. The amount of nitrated POPS was directly calculated by multiplying the amount of phosphorus by 25.

### 3.4. Mass Spectrometry Conditions

Analysis of nitrated and nitroxidized products of POPS was carried out in a negative-ion mode in an LXQ linear ion trap (LIT) mass spectrometer (ThermoFinnigan, San Jose, CA, USA), as previously described [15,16]. Nitrated POPS (aliquot of 4 µg), dissolved in methanol (2:100, *v/v*), were introduced through direct infusion, and the ESI conditions were as follows: flow rate of 8 µL min^-1^; electrospray voltage of 4.7 kV; capillary temperature of 275 °C and the sheath gas flow of 8 units. An isolation width of 1 Da was used with a 30 ms activation time for MS/MS experiments. Full scan MS spectra and MS/MS spectra were acquired with a 50 ms and 200 ms maximum ion injection time, respectively. Low energy CID-MS/MS experiments were conducted using normalized collision energy^TM^, between 20 and 30 (arbitrary units) for MS/MS. Data acquisition and results treatment were carried out with an Xcalibur Data System (version 2.0, ThermoFinnigan, San Jose, CA, USA).

High-mass-resolving ESI-MS used for the accurate mass measurements and HCD-MS/MS experiments were conducted in a Q-Exactive® hybrid quadrupole Orbitrap® mass spectrometer (Thermo Fisher Scientific, Bremen, Germany). The instrument was operated in the negative-ion mode, with a spray voltage at 2.7 kV, and interfaced with a HESI II ion source. Nitrated POPS were diluted from of 1 mg mL^−1^ stock solutions (in chloroform) using MeOH to a final concentration of 4 µg mL^−1^. The analysis were performed through direct infusion of the prepared solutions at a flow rate of 12 µL min^−1^ into the ESI source, and the operating conditions were as follows: Sheath gas (nitrogen) flow rate 5 (arbitrary units); auxiliary gas (nitrogen) 1 (arbitrary units); capillary temperature 250 °C, and S-lens RF level 50. The acquisition method was set with a full scan and resolution of 70,000, the *m/z* ranges were set to 100–1500 in negative and positive ion mode during full-scan experiments. The automatic gain control (AGC) target was set at 3 × 10^6^ and the maximum injection time (IT) was 250 ms. MS spectra were acquired during 30 s. The Q-Exactive system was tuned and calibrated using peaks of known mass from a calibration solution (Thermo Scientific, San Jose, CA, USA) to achieve a mass accuracy of <5 ppm RMS. Spectra were analyzed using the acquisition software XCalibur ver. 3.0 (Thermo Scientific). In order to obtain the product ion spectra of the nitrated derivatives of POPS and PLPS during ESI-MS experiments, the selected precursor ions were isolated by the quadrupole and sent to the higher-energy collision dissociation (HCD) cell for fragmentation via the C-trap. In the MS/MS mode, the mass resolution of the Orbitrap analyzer was set at 70,000, AGC target 3 × 10^6^, maximum IT 250 ms, isolation window 1.0 *m/z*, and normalized collision energy (NCE) was 25 (arbitrary units). Nitrogen was also used as collision gas. All experiments were repeated at least three times on different days.

### 3.5. Reverse Phase High-Performance Liquid Chromatography/Tandem Mass Spectrometry

The nitrated and nitroxidized products of POPS were separated and identified by HPLC-ESI-MS and characterized by HPLC-ESI-MS/MS, as previously described [16]. These experiments were performed on a Waters Alliance 2690 HPLC system (Mildford, MA, USA) coupled online to the LXQ linear ion trap (LIT) mass spectrometer (ThermoFinnigan, San Jose, CA, USA). An aliquot of 60 μg of each nitrated mixture (previously dissolved in chloroform, transferred to an Eppendorf tube and dried under a nitrogen stream) was diluted in 40% of mobile phase B (30 μL), and then filtered using a PVDF Millex-GV syringe filter with a 0.22 µm pore size (Millipore, Billerica, MA, USA). Volumes of 5 μL of each diluted and filtered nitrated mixture were introduced into a Discovery Bio Wide Pore C5 column (15 cm × 0.5 mm, 5 μm particle size; Supelco, Bellefonte, PA, USA), using a flow rate of 16 μL min^-1^. The mobile phase A consisted of water with 5% acetonitrile and 0.1% formic acid. The mobile phase B consisted of acetonitrile, with 0.1% formic acid. The mobile phase gradient was programmed as follows: initial conditions were 40% of B; 0–35 min, linear increase gradient to 60% of B; 35–45 min linear increase gradient to 100% of B held in isocratic mode for 10 min; 55–60 min linear gradient to 40% of B in order to brought back to the mobile phase to the initial conditions and held in isocratic mode for 5 min allowing to equilibrate the column until the next injection. The flow was then redirected to the LIT mass spectrometer using a homemade flow-splitter. The LIT mass spectrometer was operated both in the positive and the negative ion modes. Typical ESI conditions were as follows: Electrospray voltage of 4.7 kV in the negative-ion mode and 5 kV in the positive-ion mode; capillary temperature, 275 °C; and sheath gas (nitrogen) flow of 25 (arbitrary units). To obtain the product-ion spectra of the major components during LC experiments, cycles consisting of one full scan mass spectrum (*m/z* 100–1700), and three data-dependent MS/MS scans were repeated continuously throughout the experiments with the following dynamic exclusion settings: repeat count 3; repeat duration 30 s; exclusion duration 45 s. An isolation with of 0.5 Da was used with a 30 ms activation time for MS/MS experiments using helium as the collision gas. The normalized collision energy was 27 (arbitrary units). Data acquisition and processing were carried out on an Xcalibur data system (version 2.0).

### 3.6. DPPH^●^ Assay

A microplate DPPH^●^ method was used, as previously described [17,38]. DPPH^●^ method was tested for non-modified POPS or nitrated POPS. A stock solution of DPPH^●^ in ethanol (250 µmol/L) was prepared and diluted to obtain a working solution with an absorbance values of 0.9 measured at 517 nm, obtained using an UV-Vis spectrophotometer (Multiskan GO 1.00.38, Thermo Scientific) controlled by SkanIT software version 3.2 (Thermo Scientific™). In order to evaluate the stability of the radical upon reaction time, the absorbance of DPPH^●^ in the absence of antioxidant species (blank) was monitored at 517 nm every 5 min after the beginning of the reaction at room temperature, providing an absorbance decrease of 5% after 120 min. For evaluation of radical scavenging potential, 150 µL of Trolox standard solutions (between 5 and 75 µmol/L in ethanol) and non-modified POPS or nitrated POPS (75, 150 and 300 µg/mL in ethanol) were placed in each well of the 96-Well flat-bottom UV transparent microplates, followed by addition of 150 µL of DPPH^●^ in ethanol. The DPPH^●^ scavenging activity of standards and samples was monitored at 517 nm every 5 min after the beginning of reaction during 120 min, at room temperature. The experiments were performed in triplicate. The antioxidant activity of the tested samples, expressed as a percentage of inhibition of DPPH^●^ radical, was calculated (60 and 120 min) using the following equation:

% inhibition = (Abs_DPPH_^●^ − Abs_sample_)/Abs_DPPH_^●^) × 100 (1)

The IC25 values (concentration of sample that induces a reduction of 25% in the initial DPPH^●^ radical) after 120 min of reaction were calculated by linear regression from the concentration of sample versus percentage of inhibition. The results were also expressed as Trolox equivalent (TE, µmol Trolox/ g of sample). The equation used was:

TE = IC25 Trolox (µmol/L) × 1000/IC25 of sample (µg/mL)(2)

### 3.7. ABTS^●+^ Assay

A microplate ABTS^●+^ method was used, as previously described [17,39]. The ABTS^●+^ method was tested for non-modified POPS or nitrated POPS. The ABTS^●+^ radical cation solution was prepared by mixing equal volumes of an ABTS stock solution (7 mmol/L in water) with potassium persulfate (2.45 mmol/L in water). This mixture was kept for 12–16 h at room temperature in the dark and further diluted in acetate buffer (pH 4.6, 50 mmol/L) in order to achieve an absorbance value of 0.9 at 734 nm, acquired using an UV-Vis spectrophotometer (Multiskan GO 1.00.38, Thermo Scientific, Hudson, NH, USA) controlled by SkanIT software version 3.2 (Thermo Scientific™). In order to evaluate the stability of the radical upon reaction time, the absorbance of ABTS^●+^ in the absence of antioxidant species (blank) was monitored, and the absorbance decrease observed was 5% after 120 min of reaction. For evaluation of radical scavenging potential, 150 µL of Trolox standard solutions (between 5 and 75 µmol/L in ethanol) and non-modified POPS or nitrated POPS (75, 150, and 300 µg/mL in ethanol) were added to the 96-Well flat-bottom UV transparent microplates, followed by addition of 150 µL of ABTS^●+^ solution. The ABTS^●+^ scavenging activity of standards and samples was monitored at 734 nm every 5 min after the beginning of reaction during 120 min at room temperature. The experiments were performed in triplicate. The antioxidant activity of the tested samples expressed as a percentage of inhibition of ABTS^●+^ radical was calculated at specific time-points (60 and 120 min) using the following equation:

% inhibition = ((Abs_ABTS_^●+^ − Abs_sample_)/Abs_ABTS_^●+^) × 100 (3)

The IC50 values (concentration of sample that induces the reduction of ABTS^●+^ radical to 50%) after 120 min of reaction were calculated by linear regression from the concentration of sample versus percentage of inhibition. The results were also expressed as Trolox equivalent (TE, µmol Trolox/g of sample). The equation used was:

TE = IC50 Trolox (µmol/L) × 1000/IC50 of sample (µg/mL)(4)

## 4. Conclusions

Liquid chromatography coupled with the tandem mass spectrometry experiments performed in this study allowed to perform a broad characterization of the fragmentation profile of nitrated and nitroxidized derivatives of POPS. The information gathered on the typical neutral loss for each modification will be useful for lipidomic-targeted analysis of these nitrated and nitroxidized PS products in complex biological samples. Nevertheless, the confirmation of the final structure should be further accomplished by using complementary infrared and nuclear magnetic resonance analysis. Moreover, this study has identified the potential biological role of nitrated POPS, namely as a scavenging agent. Therefore, the present study contributes to the ongoing effort concerning not only the identification of nitrated phospholipids but also their potential biological roles in biological systems. Nevertheless, further research in this field is needed to provide new data on phospholipid nitration and particularly nitrated phosphatidylserine in biological conditions, and its relevance both in physiological and pathological conditions.

## Figures and Tables

**Table 1:** Neutral losses and product ions observed in the ESI-MS/MS spectra acquired in the Q-Exactive Orbitrap mass spectrometer (HCD) of the nitrated and nitroxidized derivatives formed after reaction between NO_2_BF_4_ and POPS, with the proposed identification, calculated and observed mass, error, and formula. Error (ppm) = (Observed *m/z* − Calculated *m/z*)/Calculated *m/z*) × 1 × 10^6^.

Neutral Losses	Proposed Precursor Ion Identification	Calculated *m/z*	Observed *m/z*	Error (ppm)	Formula
Precursor ion	[POPS + 29 u − H]^−^	789.5030	789.5033	0.3800	C_40_H_74_N_2_O_11_P
	NO-POPS				
Product ions					
87 u	-C_3_H_5_NO_2_	702.4701	702.4710	1.2812	C_37_H_69_NO_9_P
--	[NO-OA − H]^−^	310.2382	310.2390	2.5787	C_18_H_32_NO_3_
--	[(NO-OA) − HNO − H]^−^	279.2324	279.2332	2.8650	C_18_H_31_O_2_
--	R_1_COO^−^	255.2324	255.2330	2.3508	C_16_H_31_O_2_
--	C_3_H_6_O_5_P^−^	152.9953	152.9948	−3.2681	C_3_H_6_O_5_P
Precursor ion	[POPS + 45 u − H]^−^	805.4979	805.4986	0.8690	C_40_H_74_N_2_O_12_P
	NO_2_-POPS				
Product ions					
47 u	-HNO_2_	758.4972	758.4982	1.3184	C_40_H_73_NO_10_P
87 u	-C_3_H_5_NO_2_	718.4659	718.4665	0.8351	C_37_H_69_NO_10_P
134 u (47 + 87)	-(C_3_H_5_NO_2_ + HNO_2_)	671.4652	671.4659	1.0425	C_37_H_68_O_8_P
--	[NO_2_-OA − H]^−^	326.2331	326.2339	2.3480	C_18_H_32_NO_4_
--	[(NO_2_-OA) − HNO_2_ − H]^−^	279.2324	279.2330	2.1487	C_18_H_31_O_2_
--	R_1_COO^−^	255.2324	255.2329	1.9590	C_16_H_31_O_2_
--	C_3_H_6_O_5_P^−^	152.9953	152.9948	−3.2681	C_3_H_6_O_5_P
Precursor ion	[POPS + 61 u − H]^−^	821.4929	821.4940	1.3390	C_40_H_74_N_2_O_13_P
	(NO_2_)O-POPS				
Product ions					
47 u	-HNO_2_	774.4921	774.4928	0.9038	C_40_H_73_NO_11_P
87 u	-C_3_H_5_NO_2_	734.4608	734.4620	1.6339	C_37_H_69_NO_11_P
134 u (47 + 87)	-(C_3_H_5_NO_2_ + HNO_2_)	687.4601	687.4610	1.3092	C_37_H_68_O_9_P
--	[(NO_2_)O-OA − H]^−^	342.2281	342.2287	1.7532	C_18_H_32_NO_5_
--	[(NO_2_)O-OA) − HNO_2_ − H]^−^	295.2273	295.2279	2.0323	C_18_H_31_O_3_
--	R_1_COO^−^	255.2324	255.2330	2.3508	C_16_H_31_O_2_
--	C_3_H_6_O_5_P^−^	152.9953	152.9948	-3.2681	C_3_H_6_O_5_P
Precursor ion	[POPS + 74 u − H]^−^	834.4881	834.4883	0.2397	C_40_H_73_N_3_O_13_P
	(NO_2_)(NO)-POPS				
Product ions					
87 u	-C_3_H_5_NO_2_	747.4561	747.4547	−1.8730	C_37_H_68_N_2_O_11_P
134 u (47 + 87)	-(C_3_H_5_NO_2_ + HNO_2_)	700.4553	700.4562	1.2849	C_37_H_67_NO_9_P
--	[(NO_2_)(NO)-OA − H]^−^	355.2233	355.2255	6.1933	C_18_H_31_N_2_O_5_
--	[(NO_2_)(NO)-OA) − HNO_2_ − H]^−^	308.2226	308.2234	2.5955	C_18_H_30_NO_3_
--	R_1_COO^−^	255.2324	255.2329	1.9590	C_16_H_31_O_2_
--	C_3_H_6_O_5_P^−^	152.9953	152.9948	−3.2681	C_3_H_6_O_5_P
Precursor ion	[POPS + 77 u – H]^−^	837.4878	837.4866	−1.4329	C_40_H_74_N_2_O_14_P
	(NO_2_)2O-POPS				
Product ions					
47 u	-HNO_2_	790.4870	790.4866	−0.5060	C_40_H_73_NO_12_P
87 u	-C_3_H_5_NO_2_	750.4557	750.4569	1.5990	C_37_H_69_NO_12_P
134 u (47 + 87)	-(C_3_H_5_NO_2_ + HNO_2_)	703.4550	703.4548	−0.2843	C_37_H_68_O_10_P
--	[(NO_2_)2O-OA − H]^−^	358.2230	358.2239	2.6129	C_18_H_32_NO_6_
--	[(NO_2_)2O-OA) − O_2_ − H]^−^	326.2331	326.2338	2.1457	C_18_H_32_NO_4_
--	[(NO_2_)2O-OA) − HNO_2_ − H]^−^	311.2222	311.2230	2.5705	C_18_H_31_O_4_
--	R_1_COO^−^	255.2324	255.2329	1.9590	C_16_H_31_O_2_
--	C_3_H_6_O_5_P^−^	152.9953	152.9948	−3.2681	C_3_H_6_O_5_P
Precursor ion	[POPS + 90 u − H]^−^	850.4830	850.4835	0.5879	C_40_H_73_N_3_O_14_P
	(NO_2_)_2_-POPS				
Product ions					
47 u	-HNO_2_	803.4823	803.4825	0.2489	C_40_H_72_N_2_O_12_P
87 u	-C_3_H_5_NO_2_	763.4510	763.4517	0.9300	C_37_H_68_N_2_O_12_P
134 u (47 + 87)	-(C_3_H_5_NO_2_ + HNO_2_)	716.4503	716.4513	1.3958	C_37_H_67_NO_10_P
181 u (94 + 87)	-(C_3_H_5_NO_2_ + 2HNO_2_)	669.4495	669.4505	1.4938	C_37_H_66_O_8_P
--	[(NO_2_)_2_-OA − H]^−^	371.2182	371.2191	2.4245	C_18_H_31_N_2_O_6_
--	[((NO_2_)_2_-OA) − HNO_2_ − H]^−^	324.2175	324.2182	2.1590	C_18_H_30_NO_4_
--	[((NO_2_)_2_-OA) − 2HNO_2_ 2013 H]^−^	277.2168	277.2174	2.1644	C_18_H_29_O_2_
--	R_1_COO^−^	255.2324	255.2329	1.9590	C_16_H_31_O_2_
--	C_3_H_6_O_5_P^−^	152.9953	152.9948	−3.2681	C_3_H_6_O_5_P

**Table 2:** Typical neutral losses observed in tandem mass spectra of nitrated and nitroxidized derivatives of POPS.

		Mass Shift to Unmodified PS	Typical Neutral Losses in MS/MS
**Nitrated Derivatives**			
NO-PS	Nitroso	+29 u	−31 u (HNO)
NO_2_-PS	Nitro	+45 u	−47 u (HNO_2_)
(NO_2_)(NO)-PS	Nitronitroso	+74 u	−31 u (HNO); −47 u (HNO_2_)
(NO_2_)_2_-PS	Dinitro	+90 u	−47 u (HNO_2_); −94 u (2HNO_2_)
**Nitroxidized Derivatives**			
(NO)O-PS	Nitrosohydroxy	+45 u	−31 u (HNO)
(NO_2_)O-PS	Nitrohydroxy	+61 u	−47 u (HNO_2_)
(NO_2_)2O-PS	Nitrohydroperoxy	+77 u	−47 u (HNO_2_); −32 u (O_2_)
**Product Ions**		**Typical Modified Carboxylate Anions in MS/MS**	
[NO-OA − H]^−^	NO-PS	*m/z* 310.2	
[(NO-OA) − HNO − H]^−^		*m/z* 279.2	
[NO_2_-OA − H]^−^	NO_2_-PS	*m/z* 326.2	
[(NO_2_-OA) − HNO_2_ − H]^−^		*m/z* 279.2	
[(NO_2_)O-OA − H]^−^	(NO_2_)O-PS	*m/z* 342.2	
[(NO_2_)O-OA) − HNO_2_ − H]^−^		*m/z* 295.2	
[(NO_2_)(NO)-OA − H]^−^	(NO_2_)(NO)-PS	*m/z* 355.2	
[(NO_2_)(NO)-OA) − HNO_2_ − H]^−^		*m/z* 308.2	
[(NO_2_)2O-OA − H]^−^	(NO_2_)2O-PS	*m/z* 358.2	
[((NO_2_)2O-OA) − O_2_ − H]^−^		*m/z* 326.2	
[((NO_2_)2O-OA) − HNO_2_ − H]^−^		*m/z* 311.2	
[(NO_2_)_2_-OA − H]^−^	(NO_2_)_2_-PS	*m/z* 371.2	
[((NO_2_)_2_-OA) − HNO_2_ − H]^−^		*m/z* 324.2	
[((NO_2_)_2_-OA) − 2HNO_2_ − H]^−^		*m/z* 277.2	

## Supplementary Materials

The following are available online, Figure S1: ESI-MS/MS spectra obtained in Linear Ion Trap of the [M − H]^−^ ions at *m/z* 789.3 corresponding to [POPS + 29 u − H]^−^ (A), and at *m/z* 805.2 corresponding to [POPS + 45 u − H]^−^ (B). MS^3^ of ions at *m/z* 702.3 (A1) and *m/z* 718.3 (B1) that correspond, respectively, to [POPS + 29 u − 87 u − H]^−^ and [POPS + 45 u − 87 u − H]^−^ were also shown., Figure S2: ESI-MS/MS spectra obtained in Linear Ion Trap of [M − H]^−^ ions at *m/z* 834.6 corresponding to [POPS + 74 u − H]^–^ (A), and at *m/z* 850.3 corresponding to [POPS + 90 u − H]^–^ (B). MS^3^ of ions at *m/z* 747.3 (A1) assigned as [POPS + 74 u − 87 u − H]^–^, at *m/z* 763.3 (B1) that correspond to [POPS + 90 u – 87 u – H]^–^, and at *m/z* 803.1 attributed to [POPS + 90 u − HNO_2_ − H]^−^ were also shown., Figure S3: ESI-MS/MS spectra obtained in Linear Ion Trap of [M − H]^−^ ions at *m/z* 821.4 corresponding to [POPS + 61 u − H]^–^ (A), and at *m/z* 837.3 corresponding to [POPS + 77 u − H]^–^ (B). MS^3^ of ions at *m/z* 734.3 (A1) assigned as [POPS + 61 u − 87 u − H]^–^, and at *m/z* 750.3 (B1) assigned as [POPS + 77 u − 87 u − H]^–^ were also shown, Figure S4: Percentage of inhibition of DPPH^●^ and ABTS^●+^ radicals obtained in the presence of non-modified POPS (37.5, 75 and 150 µg/mL) after 120 min of reaction.
