# Supplementary material for: Profile of Phosphatidylserine Modifications under Nitroxidative Stress Conditions Using a Liquid Chromatography-Mass Spectrometry Based Approach
Source: Molecules. 2018 Dec 29;24(1):107. doi: 10.3390/molecules24010107 (PMC6337642; doi:10.3390/molecules24010107)
Supplement: Supplementary file 1 [file molecules-24-00107-s001.pdf]

Profile of Phosphatidylserine Modifications under Nitroxidative Stress Conditions Using a Liquid Chromatography-Mass Spectrometry Based Approach

Bruna Neves [1], Pedro Domingues [1], Maria Manuel Oliveira [2], Maria do Rosário Domingues [1,3] and Tânia Melo [1,*]

[1] Mass Spectrometry Centre, UI QOPNA, Chemistry Department, University of Aveiro, 3810-193 Aveiro, Portugal; brunafbneves@gmail.com (B.N.); p.domingues@ua.pt (P.D.); mrd@ua.pt (M.R.D.)
[2] Chemistry Department, University of Trás-os-Montes e Alto Douro, 5000-801 Vila Real, Portugal; mmso@utad.pt
[3] Biology Department & CESAM & ECOMARE, University of Aveiro, 3810-193 Aveiro, Portugal
* Correspondence: taniamelo@ua.pt; Tel.: +351 23508

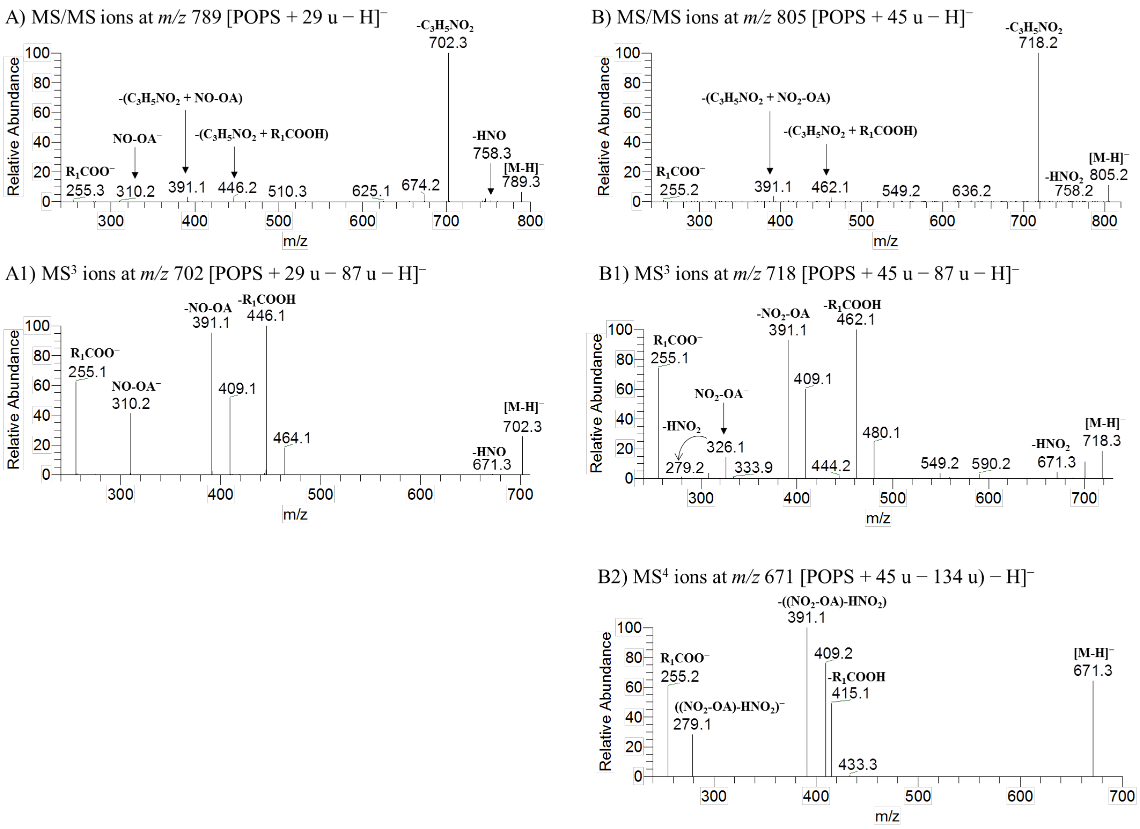

Figure S1. ESI-MS/MS spectra obtained in Linear Ion Trap of the $[M - H]^-$ ions at *m/z* 789.3 corresponding to $[POPS + 29\ u - H]^-$ (A), and at *m/z* 805.2 corresponding to $[POPS + 45\ u - H]^-$ (B). MS^3 of ions at *m/z* 702.3 (A1) and *m/z* 718.3 (B1) that correspond, respectively, to $[POPS + 29\ u - 87\ u - H]^-$ and $[POPS + 45\ u - 87\ u - H]^-$ were also shown.

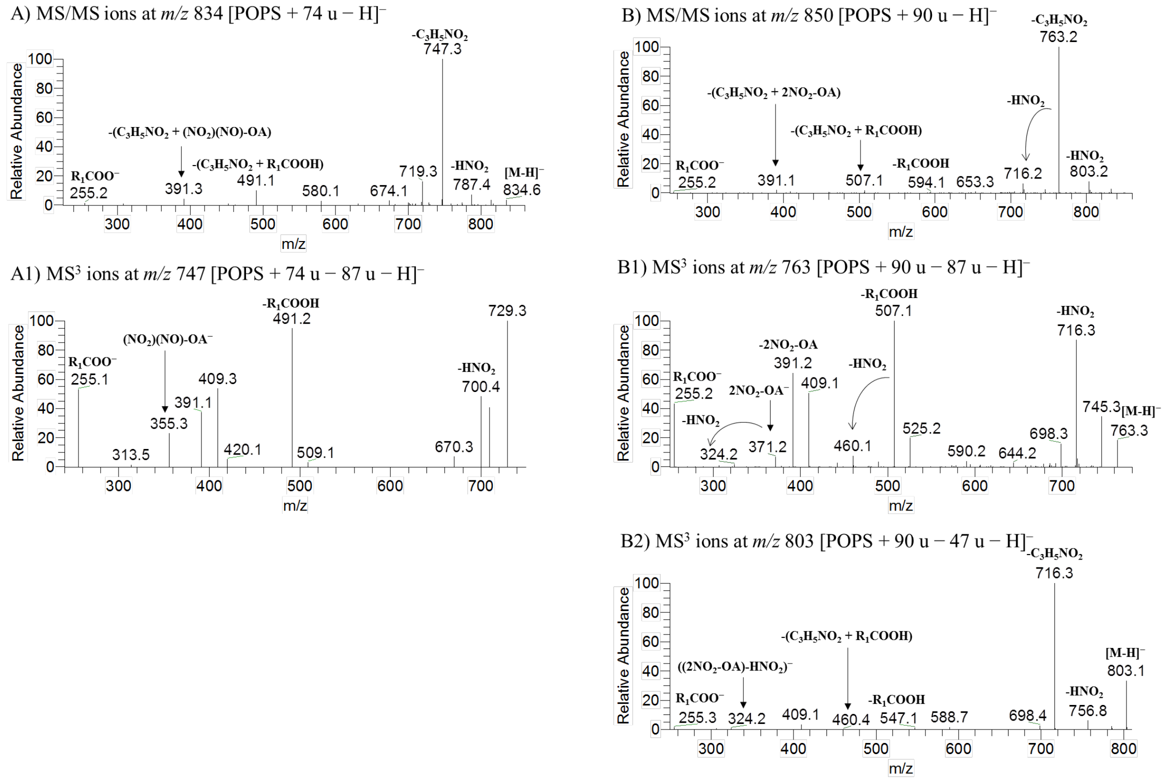

Figure S2. ESI-MS/MS spectra obtained in Linear Ion Trap of [M − H]⁻ ions at *m/z* 834.6 corresponding to [POPS + 74 u – H]⁻ (A), and at *m/z* 850.3 corresponding to [POPS + 90 u – H]⁻ (B). MS³ of ions at *m/z* 747.3 (A1) assigned as [POPS + 74 u – 87 u – H]⁻, at *m/z* 763.3 (B1) that correspond to [POPS + 90 u – 87 u – H]⁻, and at *m/z* 803.1 attributed to [POPS + 90 u – HNO_2 – H]⁻ were also shown.

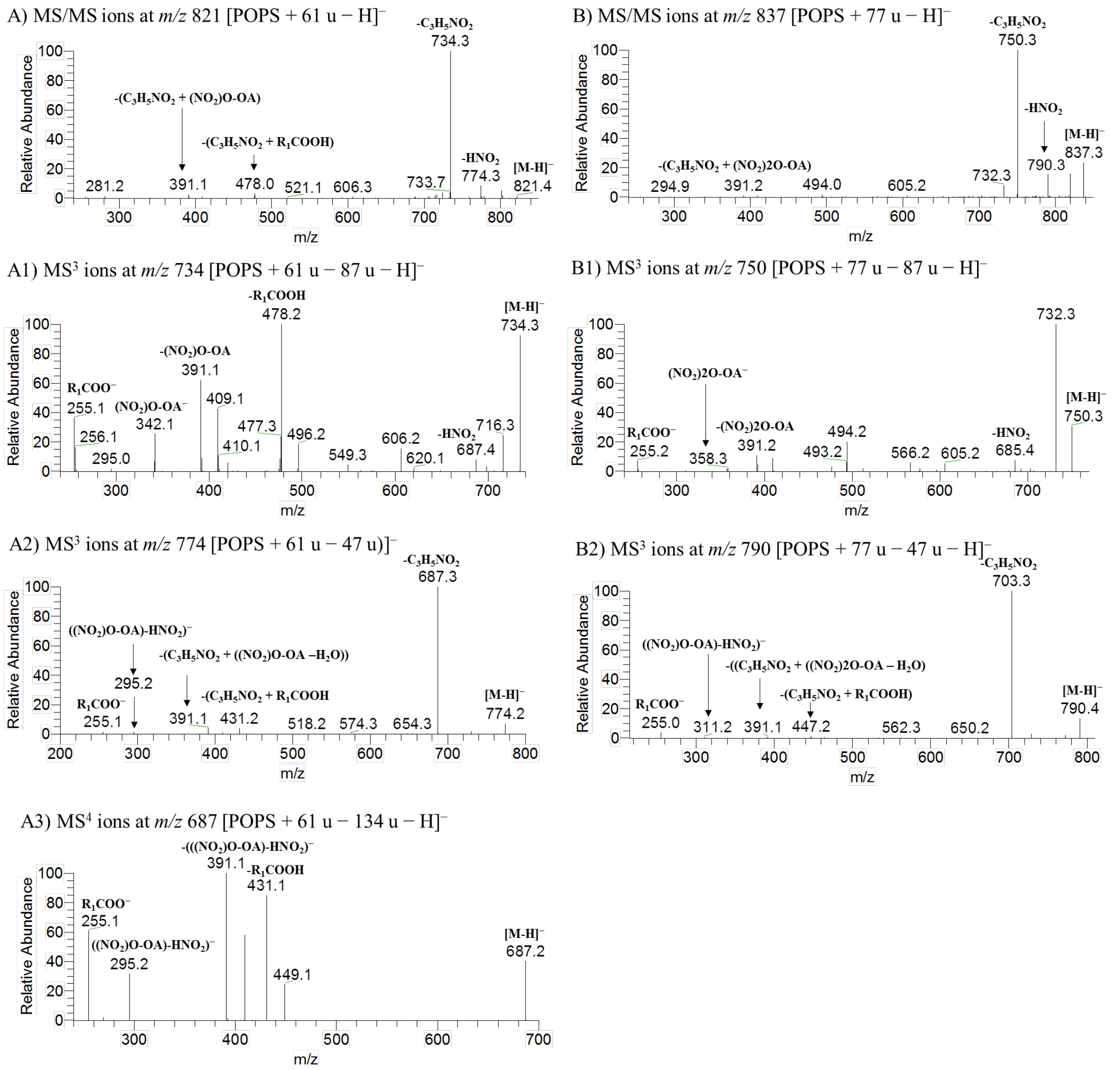

Figure S3. ESI-MS/MS spectra obtained in Linear Ion Trap of [M − H]− ions at *m/z* 821.4 corresponding to [POPS + 61 u – H]− (A), and at *m/z* 837.3 corresponding to [POPS + 77 u – H]− (B). MS³ of ions at *m/z* 734.3 (A1) assigned as [POPS + 61 u – 87 u – H]− , and at *m/z* 750.3 (B1) assigned as [POPS + 77 u – 87 u – H]− were also shown.

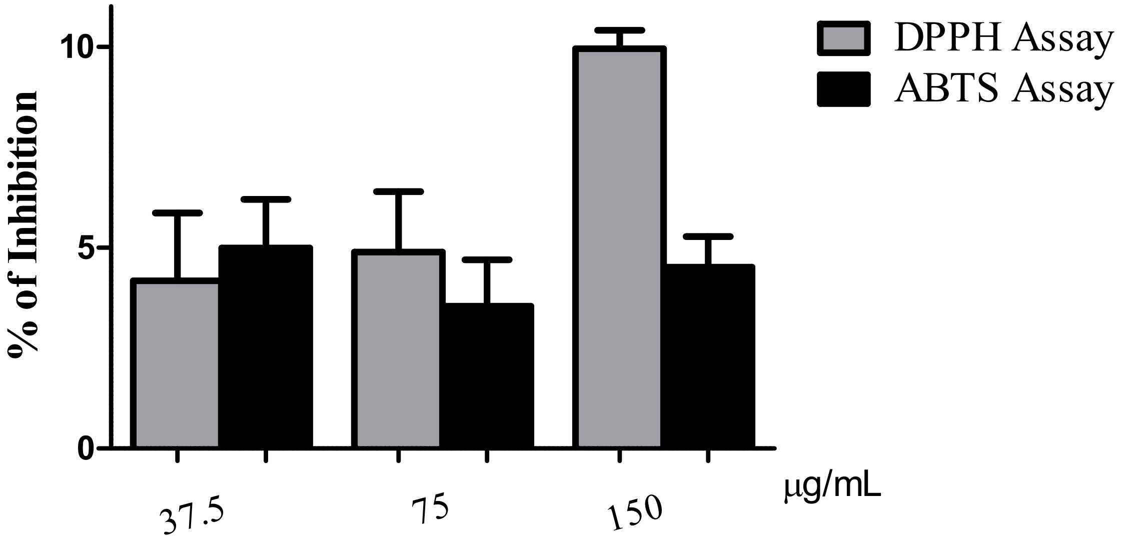

Figure S4. Percentage of inhibition of DPPH• and ABTS•+ radicals obtained in the presence of non-modified POPS (37.5, 75 and 150 μg/mL) after 120 min of reaction.
